# Enhancing Removal of Pollutants by Combining Photocatalysis and Photo-Fenton Using Co, Fe-Doped Titanate Nanowires

**DOI:** 10.3390/ma16052051

**Published:** 2023-03-01

**Authors:** B. T. Barrocas, R. Osawa, M. Conceição Oliveira, O. C. Monteiro

**Affiliations:** 1 Centro de Química Estrutural, Institute of Molecular Sciences, Departamento de Química e Bioquímica, Faculdade de Ciências, Universidade de Lisboa, Campo Grande, 1749-016 Lisboa, Portugal; 2 FT-ICR and Structural Mass Spectrometry Laboratory, MARE—Marine and Environmental Sciences Centre, Faculdade de Ciências, Universidade de Lisboa, 1749-016 Lisboa, Portugal; 3 Centro Química Estrutural, Institute of Molecular Sciences, Instituto Superior Técnico, Universidade de Lisboa, 1049-001 Lisboa, Portugal

**Keywords:** nanostructured materials, semiconductors, optical spectroscopy, electronic properties, oxidation

## Abstract

Aiming to improve their photocatalytic performance, titanate nanowires (TNW) were modified by Fe and Co (co)-doping, FeTNW, CoTNW and CoFeTNW samples, using a hydrothermal methodology. XRD characterization agrees with the existence of Fe and Co in the lattice structure.and the existence of Co^2+^ together with the presence of Fe^2+^ and Fe^3+^ in the structure was confirmed by XPS. The optical characterization of the modified powders shows the impact of the *d*–*d* transitions of both metals in the absorption properties of TNW, mainly in the creation of additional 3*d* energetic levels within the prohibited zone. The effect of the doping metal(s) in the recombination rate of photo-generated charge carriers suggests a higher impact of Fe presence when compared to Co. The photocatalytic characterization of the prepared samples was evaluated via the removal of acetaminophen. Furthermore, a mixture containing both acetaminophen and caffeine, a well-known commercial combination, was also tested. CoFeTNW sample was the best photocatalyst for the degradation of acetaminophen in both situations. A mechanism for the photo-activation of the modified semiconductor is discussed and a model proposed. It was concluded that both Co and Fe are essential, within the TNW structure, for the successful removal of acetaminophen and caffeine.

## 1. Introduction

Nowadays, the continuous discharge of pharmaceuticals and personal care products (PPCPs) in the environment and the consequent negative impact is a worldwide recognized problem. PPCPs comprises antibiotics, anti-inflammatories, antiseptics, analgesics, antidepressants, stimulants (such as caffeine), cosmetics, excipients, essences, parabens, fragrances, disinfectants, and sunscreens. The resistance of such emergent pollutants to conventional wastewater treatments causes a continuous raising level of toxicity that are incessantly impose to biota. As these products are not easily removed by conventional oxidative processes, they have been accumulating in the environment. Therefore, they can reach groundwater systems and subsequently they can reach water reservoirs [1,2]. In fact, some of these emergent pollutants have already been traced out in lakes, rivers, and even on bottled waters [3].

To face this dramatic problem, several investigations have been focussed on superior and cost-effective solutions to treat these effluents and to reduce the negative effect of their discharge in the environment. In this context, advanced oxidation processes (AOPs) are highly attractive methods to wastewaters treatment, since they are efficient, and cost-effective in the removal of a wide range of organic pollutants [4]. AOPs are eco-friendly technologies that involve the production of highly reactive and powerful redox species, like hydroxyl radical (•OH), which can degrade pollutants, by oxidation, into less harmful products [5,6]. These processes can include cavitation, photocatalytic oxidation, ozonation and Fenton’s processes. 

Typically, during the Fenton process, and because of the redox reaction of hydrogen peroxide (H_2_O_2_) and aqueous ferrous ions (Fe^2+^), there are the formation of hydroxyl radicals (•OH) [7]. Besides the use of Fe^2+^, the generation of •OH radicals through Co^2+^ and H_2_O_2_ redox reaction has been scarce reported [8].

To improve and extend the applicability of Fenton-like processes, the search for materials where Fe^2+^/Fe^3+^ can be synergetic combined with other metallic species has been explored. 

In one of the first reports, the Co^2+^/H_2_O_2_ homogeneous system was used for the removal of the basic blue dye. The removal effectiveness of the dye was strongly dependent on the H_2_O_2_ concentration [8]. Analogous conclusions were found in photo-Fenton and electro-Fenton studies, involving the use of Co^2+^ aqueous or heterogeneous catalytic combinations such as Co^2+^/Al_2_O_3_, Co^2+^/MCM-41, or Co^2+^/carbon aerogel [9]. In a different work, the isomorphic substitution of Fe^2+^ in the *magnetite* structure, by ionic Mn^2+^ or Co^2+^ induced a relevant enhancement in the catalytic performance of the correspondent Fenton-like system [10]. An increase in the Fenton-oxidation performance was also reported when small amounts of Cr were used. This positive effect was attributed to the joining effect of Fe^3+^/Fe^2+^ and Cr^2+^/Cr^3+^ redox pairs [10].

In last years, together with Fenton processes, the photo-assisted degradation of pollutants, using semiconductor nanocrystalline materials as catalysts has been a relevant research subject. Together with the efficient removal of harmful pollutants, the inactivation of antibiotic-resistant bacteria can be attained [11]. Titanate nanowires (TNW) are very fascinating materials once they hold some of the TiO_2_ nanoparticles characteristics, in a synergetic combination with some properties of layered titanates, such as photocatalytic and ion-exchange capabilities, respectively. Nevertheless, and if compared to TiO_2_, they also present high charge recombination levels and wide bandgap energy, which can limit their practical applications. Thus, the production of TNW-based materials with visible-shifted activation and/or reduced recombination rate of photo-generated charge carriers will be important achievements aiming the development of new catalysts for photo-activated redox processes. Several methodologies, including transition metals incorporation doping, hybridization, or nanocomposite combination, have been explored to answer this challenge. 

It has been recognised that pristine or modified titanate nanostructures have improved photocatalytic and significant adsorption capacity for a wide range of organic products [12,13,14,15,16,17,18,19,20]. For instance, TNW-modified with ZnS and Ag_2_S nanoparticles have demonstrated to possess interesting adsorbent and photocatalytic properties for organic pollutants removal [17]. When sensitized with organic entities, like amines, lamellar titanate nanoparticles shown high performance for PPCPs photo-assisted removal [15]. If previously sensitized with cyanocobalamin, TNW is very efficient in the degradation of rhodamine B dye [16]. Studies on the modification of TNW with Co [21], or Ru [22,23], shown that the incorporation of such metals in different structural positions (replacing Ti^4+^ in the TiO_6_ octahedra or Na^+^ in the interlayers), will causes significative alterations in their photocatalytic performances.

Recently, it was stated that Fe^3+^ doping induces a positive effect in the TiO_2_ photoactivation, especially when submitted to visible radiation [24]. Fe^3+^ act as doping agent in TiO_2_ structure, once they possess a half-filled electronic arrangement, and the Fe^3+^ modified TiO_2_ not only have a positive action in the photogenerated electrons and holes pairs recombination but also it reduces the TiO_2_ bandgap energy [25,26].

Lately, an improvement on the photocatalytic properties, conjugated with an occurrence of a photo-Fenton process, were reported for TNW particles after co-doping with Fe and Mn species [27]. The combination of Fe and Co, as dopants, aiming the synthesis of a photocatalytic active Fe/Co-doped titanate nanotubular material, to be used for water splitting, was recently reported [28]. Unfortunately, the doping process was performed by an ion-exchange methodology and the presence of segregated Fe_2_O_3_ and metallic cobalt together with the titanate nanotubular particles was described.

In line with the above described, in this work it is proposed the synthesis of TNW materials co-modified by Fe and Co doping to be used for photo-assisted pollutants removal. The effect of incorporating a metal (Co), with well-known (photo)catalytic properties, into a material with expected (photo)Fenton activity, due the Fe presence, was aimed. Cobalt was chosen because in addition to its excellent catalytic properties, it can also participate in Fenton reactions and therefore can have a dual positive effect in the photo-assisted removal process of a pollutant. After characterization, the prepared samples, containing Fe, Co and both metals, were used for the removal of acetaminophen. The removal process was studied using a combination of caffeine and acetaminophen, a commonly found commercial pharmaceutic formulation, and the intermediates formed were identified and quantified. To understand the impact of the photocatalytic degradation process and of the Fenton counterparts, the use of scavengers was required. A mechanism scheme involving a synergetic combination of heterogeneous photo-Fenton and photocatalytic degradation processes was proposed.

## 2. Materials and Methods

All reagents used during this work were of analytical or chemical grade (Aldrich, Fluka or Acros) and no purification was performed before use. 

### 2.1. M-Containing Precursors (M = Fe, Co) Synthesis

First, the preparation of a TNW precursor was performed, in accordance with a published procedure [29]. In brief, 50 mL of titanium trichloride solution (12 wt.% in 20–30 wt.% HCl, Acros) was diluted in a 1:2 ratio using HCl solution (2M). After, an ammonia aqueous solution (4 M) was added dropwise under stirring, until the complete precipitation of a solid. The suspension was kept overnight at room temperature. After, it was filtered and the solid washed with water several times. The solid was dried and stored. To obtain the Co-containing TNW samples, a nominal percentage of 1% molar Co^2+^ was added to the titanium trichloride solution during precursors synthesis [23].

The same synthesis procedure was used for the preparation of the Fe-doped precursor but in this case a different TiCl_3_ solution (12 wt.% in 20–30 wt.% HCl), from Aldrich, was used. From a previous report, contamination levels of 4 mg L^−1^ Fe and 8 mg L^−1^ Mn, in this commercial product, were confirmed. Using this Ti source and the same experimental conditions, the Mn amount incorporated in the final titanate nanoparticles was vestigial, lower than Mn/Ti = 0.008%, but a higher amount (0.14%) was observed for Fe/Ti ratio [27]. Considering this fact, only the effect of the Fe presence is discussed in this work. 

### 2.2. Pristine and Doped TNW Particles Synthesis

TNW and metal doped powders were produced in an autoclave system using previously reported protocol [21], and a schematic representation is presented in Figure S1. The temperature and time were set to 160 °C and 24 h. The pristine sample was labeled as TNW (titanate nanowires). The samples containing Fe, Co and Co-Fe were labeled as FeTNW, CoTNW and CoFeTNW, respectively. 

### 2.3. Photocatalytic Degradation Studies

Acetaminophen was used as model pollutant to study the photocatalytic ability of the TNW prepared samples, under UV-vis radiation. The catalytic photodegradation experiments were performed using 20 mg of catalyst to 150 mL of acetaminophen aqueous solution (20 ppm). To evaluate the influence of having a combination of the two pollutants, commonly found in pharmaceutic formulations, a photodegradation assay using a mixture of acetaminophen and caffeine was also performed. The photo-assisted oxidation runs were conducted in a 250 mL photo-reactor equipped with a 450 W medium-pressure mercury-vapor lamp [30]. The identification and quantification of the degradation products was reached by LC-ESI-QTOF-MS. The photocatalytic experiments were performed in duplicate for each catalyst, and an error inferior to 1.5% was obtained.

### 2.4. Hydroxyl Radical (•OH) Photocatalytic Production

Terephthalic acid (TA) was used as probe to evaluate the ability of the prepared samples for catalyze the photocatalytic production of the hydroxyl radical. TA is a natural OH• scavenger and the photo-degradation of such radical can be monitored through the formation/detection of the fluorescent 2-hydroxyterephthalic acid (HTA) [31]. The TA photocatalytic experiments were carried out using the conditions described above but using a 3 mM TA solution (freshly prepared in 0.01 M NaOH aqueous solution) and 10 mg of each solid. During irradiation, the suspensions were sampled, centrifuged, and analyzed by fluorescence spectroscopy. Photocatalytic experiments were also performed using different scavengers to quench radical’s species. Ethanol (0.5 mM) was employed as •OH scavenger, potassium oxalate (0.5 mM) as *h^+^* quencher, and benzoquinone ((0.1 mM) was added to the reaction media as O_2_^•−^ scavenger. 

### 2.5. Characterization

The obtained powdered samples were characterized by X-ray powder diffraction (XRD) using a Philips Analytical X-ray diffractometer (PW 3050/60) and the acquisition protocol described [32]. The morphology of the prepared materials was analyzed in a JEOL 200CX electron microscope (300 kV) as previously described [14]. The surface area of the samples was attained by the Brunauer-Emmett-Teller (B.E.T.) technique, using a reported protocol [22]. The optical behavior of the solid samples, mainly their diffuse reflectance spectra (DRS), were recorded in a Shimadzu UV-2600PC UV−vis spectrometer equipped with an integrating sphere (ISR 2600 plus). Without the integrating sphere accessory, this equipment was used to register the of acetaminophen and caffeine solutions absorption spectra, during the photocatalytic assays. A Kratos AXIS Ultra HAS spectrophotometer, equipped with a monochromatic Al Kα X-ray source (1486.7 eV), and with VISION and CASAXPS software, was used for XPS data acquisition and analysis, respectively. Details on data acquisition and analysis are described [22]. The amount of iron and cobalt, incorporated in the TNW structure was assessed by micro-X-ray fluorescence (XRF). The equipment used was a M4 Tornado from Bruker containing an Oxford Instruments Eclipse IV X-ray source with a Rh anode and a 250 m thick Be window (50 kV, 300 A, 150 s) coupled to a XFlash^®^silicon drift detector [27]. The emission spectra of the TA-containing solutions during irradiation were recorded on a SpexFlourolog 3-22/Tau 3, using 315 nm and 425 nm as excitation and emission energies, respectively. During irradiation, caffeine and acetaminophen containing aliquots were also analyzed on a QqTOF Impact IITM mass spectrometer equipped with an ESI source (Bruker Daltonics, Germany) and interfaced with a Dionex UltiMate^®^ 3000 RSLCnano (Thermo Fisher Scientific Inc., Waltham, MA, USA). Detailed equipment specifications and experimental protocol for calibration, data acquisition and processing are described [22].

## 3. Results and Discussion

### 3.1. Structural and Morphological Characterization

**Figure 1:**
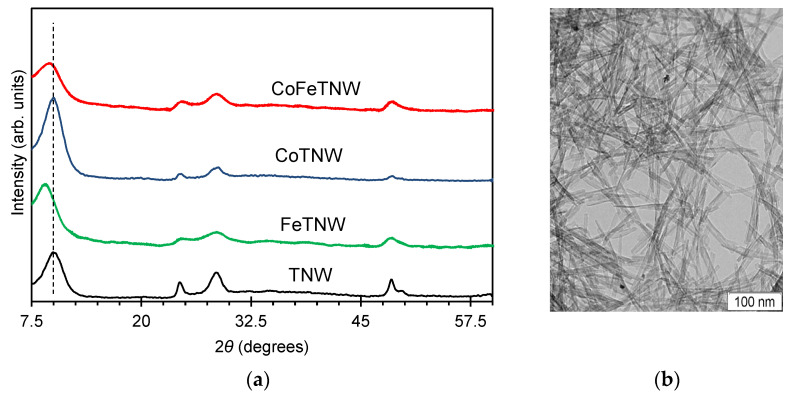
(**a**) XRD patterns of the TNW, FeTNW, CoTNW and CoFeTNW powders, (**b**) TEM image of a TNW sample.

After synthesis, the powders were analyzed by XRD and the results are presented in Figure 1. Pristine TNW powder shows typical diffraction pattern of a crystalline Na_2_Ti_3_O_7_ layered titanate structure, with 2*θ* peaks centered at 9.94°, 24.35°, 28.59°, and 48.48° [15,33,34,35]. Signs of others crystalline phases, e.g., metallic or oxide(s) phases were not identified in the diffraction patterns. The characteristic titanate lamellar structure is composed by TiO_6_ octahedra lamellas, with the diffraction peak at 2*θ* = 9.94° assigned the distance between these lamellas. The usual host of this space are Na^+^ ions, resulting from the synthesis media. 

The XRD pattern of the CoTNW sample is identical to that obtained for TNW. No shifts on the peak associated with the TiO_6_ interlayers distance at 2*θ*~10° were visualized. According to literature, this suggests that no significative Co/Na substitution has occurred, with the metal incorporation taking place mainly by replacement of Ti^4+^ by Co^2+^ ions [21,36].

For the Fe containing samples, FeTNW and CoFeTNW, a shift of the above-mentioned peak, from 2*θ* = 9.94° to 9.01° and to 9.47°, respectively, was observed. Therefore, the incorporation of metal ions between the TiO_6_ layers, via an ion-exchange procedure can be expected. This assumption agrees with other reported works describing shifts in this peak associated to Na^+^ exchange by species like H^+^, Ce^4+^ or Co^2+^ [3,14,21]. It should be noted that the decrease in this 2*θ* peak, observed for Fe,Co-containing samples, indicates that the distance between the TiO_6_ layers increases, thus being in accordance with the larger ionic radius of Fe and Co in relation to that of Na^+^ [36].

These results also agree with the hypothesis that the growth of TNW sheets is preceded by the dissolution of the precursor in the NaOH media [37,38]. Therefore, some of the Co and/or Fe ions are retained in solution and thus allowing the production of metal-containing materials, with the metallic ions in the interlayers.

The morphology of prepared TNW powders was analyzed by TEM. All samples were homogeneous and constituted by very thin and elongated nanoparticles, with a diameter of ~7 nm (Figure 1 and Figure S2). No other particles than nanowires were observed. For the doped samples, no influence of either Fe or Co incorporation on the final morphology of these materials was visualized. The quantification of the metals incorporated in the semiconductor structure, was attained by XPS and XRF analyses. The M/Ti ratio (M = metal) for all samples are presented in Table 1. The Fe/Ti ratio is identical for FeTNW and CoFeTNW powders, 0.14 and 0.15% respectively. The corresponding Co/Ti ratio is almost ten times higher than that of Fe/Ti. Identical values, 1.6 and 1.7% were obtained for CoTNW and CoFeTNW, respectively.

The characteristic extensive surface area of such semiconductor nanoparticulate materials, is one of their most attractive properties, especially if their use as catalyst is foreseeing. Thus, this parameter was assessed by B.E.T. methodology. TNW powder has a surface area of 238.10 m^2^ g^−1^, a value very similar to the one obtained for CoTNW particles, 237.52 m^2^ g^−1^. This agrees along with literature [21,39], and is consistent with a partial substitution of ionic titanium by cobalt in the TNW crystal structure, as suggested by the XRD data. Surface areas of 124.74 m^2^ g^−1^ and 180.14 m^2^ g^−1^ were obtained for FeTNW and CoFeTNW samples, respectively. 

**Figure 2:**
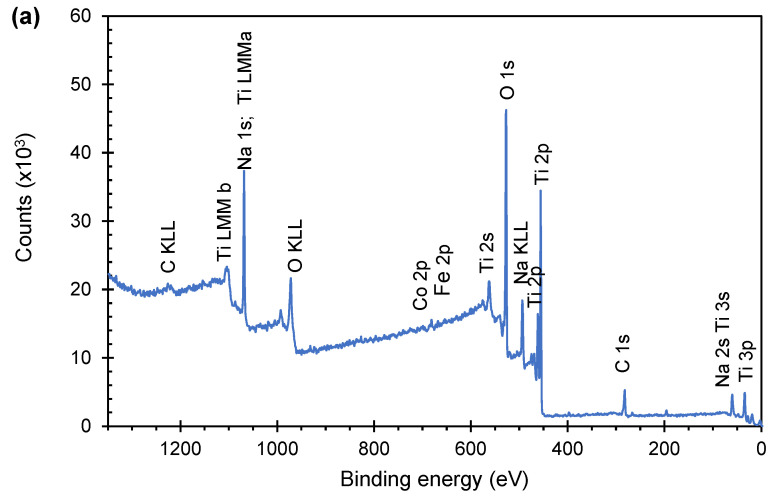

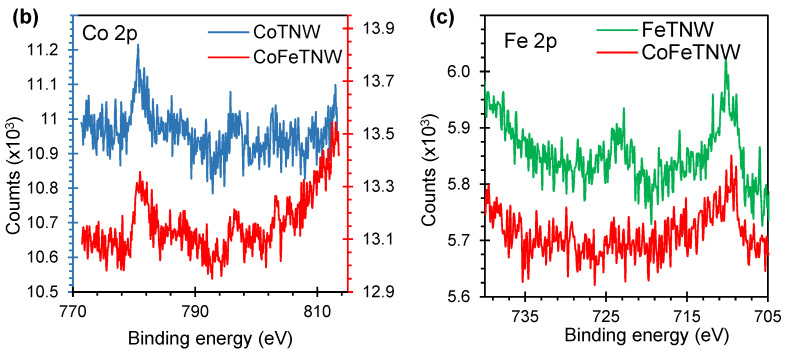
(**a**) XPS survey spectrum of the CoFeTNW sample. XPS high resolution spectra of the Co 2*p* (**b**) and Fe 2*p* (**c**) regions of the prepared samples.

The pristine and modified TNW samples were analyzed by XPS to get more information about the structural impact of the doping process, namely on the presence of Fe and Co in the lattice structure (interlayers, interstitial positions or as Ti^4+^ substituent). Figure 2a shows, as an example, the survey spectra recorded for CoFeTNW powder. In addition to the photoelectron peaks characteristics of TNW, such as Na 1*s*, Ti 2*p*, and O 1*s*, small peaks attributed to Fe 2*p* and Co 2*p* are also present. Similar profiles were obtained for all samples. However, depending on their chemical composition, the Fe and/or Co signals can also be visible. For CoFeTNW, CoTNW and FeTNW, the high-resolution spectra of the Fe 2*p* and Co 2*p* regions are shown in Figure 2b,c. 

In the XPS spectrum of TNW sample (not shown), typical Ti 2*p*_3/2_ and 2*p*_1/2_ peaks, corresponding to an octahedral coordinated Ti^4+^ state, are visible. Slightly shifts on the position of these peaks, for lower energies, were found for all the modified samples (Table S2). For Ti 2*p*_3/2_, the higher shift (0.281 eV) was observed to FeTNW, followed by CoTNW with 0.163 eV; the sample with less changes (0.147 eV) was the co-doped CoFeTNW. It is known that a shift in the position of these peaks usually translates the influence of metal addition on the electronic state of Ti element, being in this case more pronounced for Fe rather than for Co incorporation; with some of the Ti^4+^ to be substituted by Fe ions. This agrees with the previously discussed XRD data. 

The doublet splitting energies of the Ti 2*p* peaks (ΔE) was calculated and an increase on the reference value for Ti^4+^ obtained for pristine TNW (5.8 eV) was shifted to higher values for Fe and Co containing powders (Table S2), indicating, the presence of Ti^4+^/Ti^3+^. This confirms the incorporation of species with oxidation state different than 4+, and thus conducting to the formation of oxygen vacancies [21,40,41,42].

For all the TNW-based samples, the O 1*s* spectra, is dominated by a main peak at ca. 532 eV, that is due to structural oxygen. A slight contribution of the Auger peak of sodium, Na KLL should also be considered.

For Co containing samples, the Co 2*p* high-resolution spectra are present in Figure 2b. The presence of cobalt as Co^2+^ in both samples is confirmed by the existence of Co 2*p*_1/2_ and Co 2*p*_3/2_ peaks at 780.99 and 796.45 eV for CoTNW and at 780.73 and 796.33 eV, for CoFeTNW [43,44].

In the Fe 2*p* spectra (Figure 2c) there is a doublet peak at 710.1 and 723.7 eV, for CoFeTNW corresponding to the Fe 2*p*_3/2_ and 2*p*_1/2_ spin–orbital components. No significant shift on these peaks position was observed for the FeTNW sample. The existence of these peaks indicates the presence of Fe^2+^ and Fe^3+^ in the structure of both samples [27,45,46].

### 3.2. Optical Characterization

In elongated titanate nanostructures, the absorption edge at ~380 nm, corresponds to the energy required for the electron transfer from the valence band (VB), in this case O 2*p* orbital, to the conduction band (CB), that corresponds to Ti 3*d* orbital [47].

**Figure 3:**
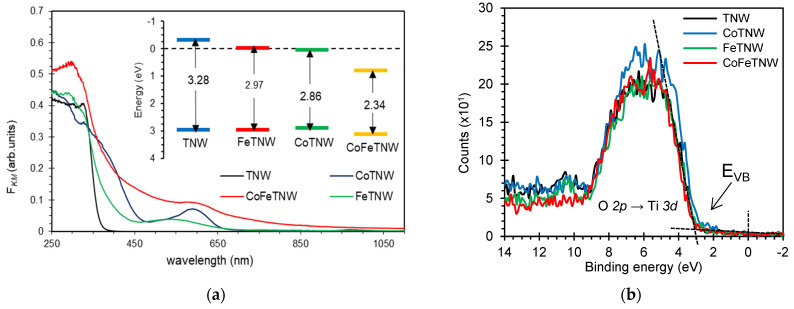
(**a**) Optical absorption with correspondent energetic diagram (as inset) and (**b**) XPS valence band spectra of the prepared samples.

Foreseeing their future application as photocatalysts, the samples were optical characterized by recording their diffuse reflectance spectra in the UV-vis range. *R* can be related with the Kubelka–Munk absorption function, *F*_KM_, by *F_KM_* (*R*) = (1 – *R*)^2^/2*R* [48]. The optical spectrum of TNW powder in Figure 3 is in accordance with literature [14]. However, new features are visible in the TNW absorption profile after Co and Fe incorporation. Thus, the spectra of such samples also exhibit an absorption band at 480–650 nm, that is typical of transition metal doped semiconductor nanoparticles; this feature arising from the *d*–*d* transitions of the electrons within the metal [49].

The optical red shift observed for Co-containing TNW particles is due to the incorporation of Co^2+^ 3*d* states in TNW crystalline structure, and their consequent splitting, that leads to the creation of lower and upper 3*d* energetic levels within the prohibited zone of the semiconductor [21,50]. Also responsible for this shift are the oxygen vacancies created due to Co^2+^/Ti^4+^ replacement [16,21].

The FeTNW sample presents a band edge that is slightly red shifted in comparison to the characteristic TNW pristine particles. This contraction can be due to the overlapping of the Fe 3*d* orbitals, with the subsequent extension of the absorption to the visible [39]. For this sample, it is clearly seen the absorption band located at 440–540 nm, that has been assigned to the *d*–*d* transitions of the metal [50,51].

The optical spectrum of the sample containing both metals and in accordance with literature [21,27], ought to show, and together with the absorption edge characteristic of TNW, the impact of the *d*–*d* transitions of both metals, Co and Fe. In fact, the optic spectrum of CoFeTNW exhibits absorption features that result agree with the presence of both Co and Fe in the TiO_6_ octahedra and in the interlayers. This data is also in accordance with XPS and XRD data. 

The bandgap energy (*E_g_*) of the prepared powders was evaluated using a reported procedure [47]. Values of 3.28 eV, 2.97 eV, 2.86 eV and 2.34 eV were obtained for *E_g_* of the TNW, FeTNW, CoTNW and CoFeTNW samples, respectively (Table S1). The incorporation of Fe and/or Co into the TNW structure, shifts the absorption edge of the pristine semiconductor of about 0.31, 0.42 and 0.94 eV towards the lower energy region, for FeTNW, CoTNW and CoFeTNW samples, respectively.

The valence band energy (*E_VB_*) was determined by using the XPS valence band spectrum (Figure 3b) and the conduction band energy (*E_CB_*) was subsequently calculated using a reported method [52]. The obtained results are presented in Table S1 and for a straightforward interpretation, they are also schematized in Figure 3a-inset.

To evaluate the effect of the doping metal(s) in the recombination of the photo-generated charge carriers, photoluminescence spectra of the samples were recorded (Figure S3). In comparison to TNW, a decrease in the intensity of the emission peak at ~425 nm was observed for Fe and Co containing powders. This agrees with the above analysis of the optical data, and it could be related to a more efficient electron-hole separation in the modified TNW materials. A comparative analysis of the PL spectra of the Fe and Co containing samples suggests a higher, and more positive, impact of the Fe presence if compared to Co, in the recombination rate reduction.

### 3.3. Photocatalytic Performance 

**Figure 4:**
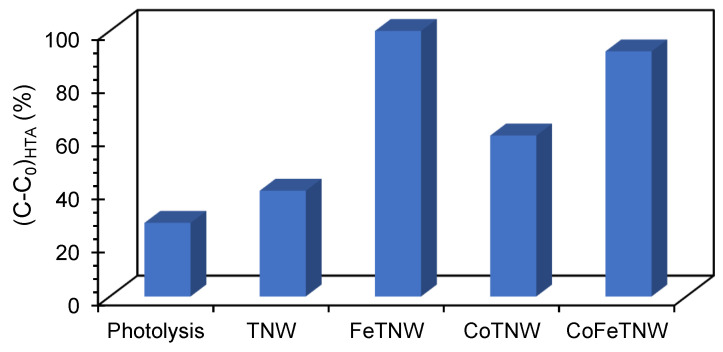
HTA concentration after 20 min of irradiation of a TA solution (3 mM, 150 mL) using the prepared samples as photocatalyst.

Since hydroxyl radical (•OH) is one of the best oxidizing agents for the photocatalytic degradation of pollutants, its photocatalytic production was monitored in the presence of the prepared samples, using terephthalic acid (TA) as probe molecule [19,31]. Despite that all the samples were catalytic for this reaction (Figure 4), FeTNW was the best photocatalyst. Comparing with photolysis, an increase of 70% and 63% on •OH production was reached using FeTNW and CoFeTNW as catalysts, respectively. Lower performances were attained by CoTNW and TNW samples, with •OH production in the range of 30% and 10%, above photolysis, respectively. Hence, it can be concluded that the presence of Fe in the TNW structure induces a higher •OH photocatalytic production rather than Co. Corroborating with this conclusion is the fact that the best photocatalysts, FeTNW and CoFeTNW, were the samples presenting the lower surface area, 124.74 and 180.14 m^2^ g^−1^, respectively.

Two main reasons can be in charge for the excellent catalytic performance of FeTNW for •OH production: (i) the relevant contribution of both heterogeneous photo-Fenton and photocatalysis processes; (ii) the comparative lower catalytic performance observed for CoFeTNW in comparision to FeTNW (these two samples have identical Fe/Ti ratio) can be justified by its *E_CB_* that prevents the O_2_ reduction to H_2_O_2_ and consequently, impedes the Fenton process occurrence.

The photocatalytic performance of the prepared samples was further evaluated for the photocatalytic removal of a worldwide used pharmaceutic: acetaminophen (ACAP). The adsorption of ACAP in the surface of the catalysts was evaluated in the dark during 60 min, previously to the irradiation period. During the photocatalytic experiments, aliquots were taken, and, after centrifugation, they analyzed by UV-vis spectroscopy and afterwards by LC-ESI-QTOF-MS.

**Figure 5:**
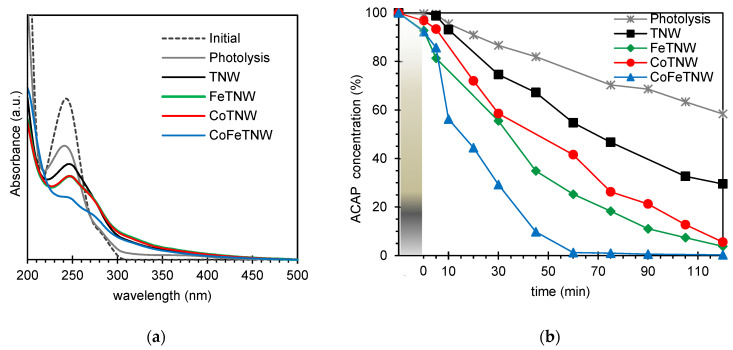
UV-vis spectra (**a**) of a 20 ppm ACAP solution after 120 min of irradiation, and (**b**) degradation profiles obtained from LC-ESI-QTOF-MS, using the prepared samples as photocatalysts (20 mg of catalyst to 150 mL of ACAP solution).

The spectra of ACAP solutions after 120 min of irradiation are shown in Figure 5. Since there are some intermediates absorbing in the same range of the parent pollutant (Figure 5a) such spectra were not used for further analysis. Therefore LC-ESI-QTOF-MS data was used for the identification and quantification of ACAP intermediates.

From Figure 5b analysis, it is visible for all samples, excepted for TNW, a slight adsorption of the pollutant during the dark period before irradiation. CoFeTNW adsorbed 7.7%, FeTNW 7.3% and CoTNW only 3.2% of the initial ACAP present in solution. All the samples revealed to be catalytic for this photo-assisted oxidation process. All the metal(s) containing TNW samples demonstrated to be better than pristine TNW particles. 

CoFeTNW was the best photocatalyst, with the complete removal of ACAP attained after 60 min of irradiation. For the same period, ACAP removal rate was 74.7% using FeTNW as catalyst, 58.4% for CoTNW and 45.3% when pristine TNW particles were used. Nevertheless, and for these 3 catalysts, a longer period, rather than 120 min, would be required if the complete degradation of ACAP is aimed. It should be noted that after 120 min of irradiation, only 41.6% of ACAP degradation was achieved via photolysis; this is the degradation rate attained after the first 10 min of irradiation, when the best photocatalyst, CoFeTNW, was used. 

It should be noted that after the first 10 min of irradiation, 43.8% of initial ACAP were degraded using the best photocatalyst, CoFeTNW; identical degradation rate was attained via photolysis but only after 120 min of irradiation.

The obtained results show and agreeing with the above discussed for hydroxyl radical production, that the surface area of the samples have no influence on their photocatalytic performance; being the Fe and Co doping the parameter responsible for the major effect.

The identification and quantification of intermediates present in solution during this process was performed and are present in Table S3. The identification of five intermediates (ACAP-1 to ACAP-5) during the irradiation period agree with a degradation pathway proposed in literature [53,54].

### 3.4. ACAP and CAF Mixture Photocatalytic Removal

**Figure 6:**
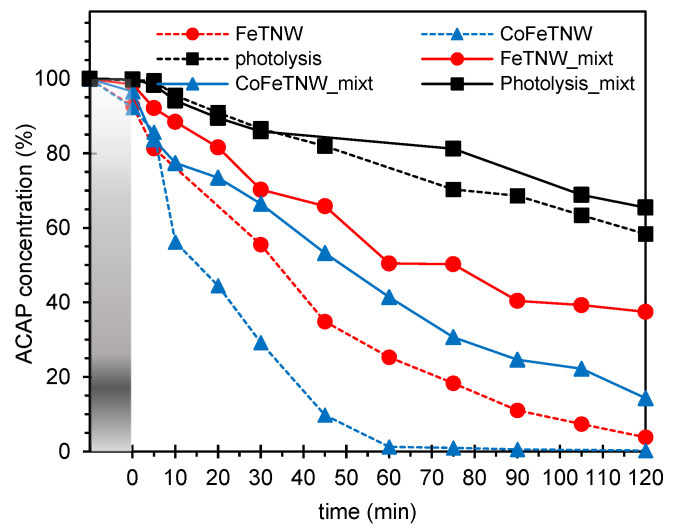
ACAP removal profiles for single and mixture solutions, during 120 min of irradiation, for photolysis and using FeTNW and CoFeTNW as catalyst photocatalysts (20 mg of catalyst to 150 mL of ACAP solution).

To better evaluate the stability of the prepared samples as future photocatalysts in real world applications, studies on the simultaneous degradation of a mixture of ACAP and caffeine (CAF) were carried on. Supported on the data above-discussed, FeTNW and CoFeTNW were the samples selected for this study. The experimental conditions used, namely the concentration of each pollutant in the mixture, the amount of catalyst and the time of irradiation (120 min) were kept unchanged. In Figure 6 are shown the degradation profiles of ACAP, when used as single and mixture solution. The best catalyst for the photo-assisted degradation of the (ACAP+CAF) mixture was CoFeTNW sample.

Despite this, it is interesting to note that ACAP overall photodegradation process is clearly (negative) influenced by CAF presence. Nevertheless, for ACAP degradation via photolysis (120 min), the mixture effect is not so obvious since degradation levels of 41.6% and 34.5% were attained for single and mixture solutions, respectively. On the other hand, and as can be visualized in Figure 6, the kinetic process associated with photolysis is considerably slower when compared to the photocatalytic experiments. This suggest that it can be very difficult to achieve the complete degradation of the pollutants only by the photolysis effect.

### 3.5. Reusability and Stability

The stability and reusability of the best photocatalyst (CoFeTNW) were tested for 4 runs of 75 min each. The photodegradation results obtained for these runs (not shown), indicate that this catalyst is stable without significant loss of catalytic activity; % of degradation between 98.89 and 99.08 were obtained. These results indicate that during these 4 cycles, no signals of surface poisoning and no release of any metallic or others species from the catalyst to the solution were observed, attesting the high stability of these samples when used as photocatalysts under UV-vis radiation. XRD analysis were performed to confirm the stability of the powder before and after being submitted to radiation and no perceptible changes or rearrangements in the microstructure were observed.

### 3.6. Scavenger’s Study 

To go further in this study and to analyze whether the ACAP photocatalytic degradation takes place via oxygen radical species (ROS), such as O_2_^•−^, •OH or via direct positive holes (*h^+^*) or electron transfer, radical scavengers like benzoquinone, ethanol, and potassium oxalate, were added to the reaction media during irradiation. Considering the photocatalytic performance data discussed above, CoFeTNW sample was chosen for proceed with this study.

**Figure 7:**
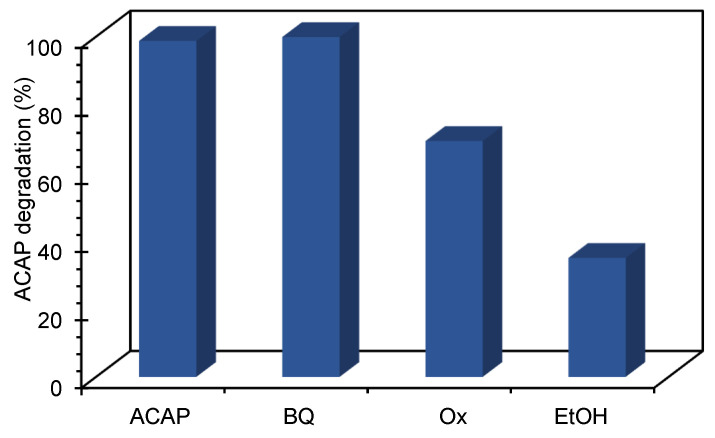
Photodegradation rate of ACAP, during 60 min of irradiation when in the presence of selected scavengers and using CoFeTNW as catalyst.

Ethanol (EtOH) and potassium oxalate (Ox), here used as •OH and *h*^+^ scavengers, respectively, were the responsible for the reduction of the ACAP degradation rate (Figure 7). Using benzoquinone, a O_2_^•−^ scavenger, no significant differences in the pollutant removal were observed. This result agrees with the hypothesis postulated in *Hydroxyl radical production* section and with the data obtained during optical characterization, attesting that for this sample no O_2_ reduction occurs due to *E_CB_* energetic constraints. Within these results, it is plausible to conclude that this pollutant degradation does not take place directly via O_2_^•−^ but •OH and *h*^+^ are the main oxidant species involved in this process. 

### 3.7. Photo-Activation Mechanism Proposal

**Figure 8:**
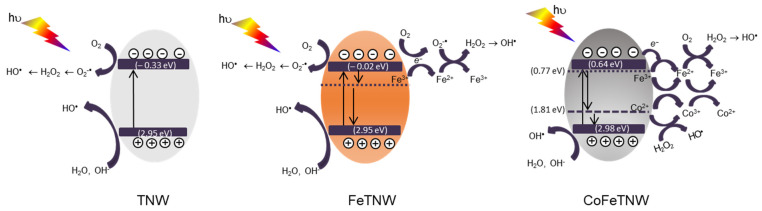
Photo-activation mechanism proposed for TNW, FeTNW and CoFeTNW hybrid nanoparticles.

Supported in the above discussed data and in reported works [55,56]. A mechanism for the light-activation of such nanomaterials is proposed in Figure 8. For transition metals modified TNW and depending on the metal(s) 3*d*-levels energy, the doping process can prompt the creation of specific energy levels inside the forbidden zone. Therefore, it has been proposed [21,22,27,57], that when a transition metal doped TNW is exposed to radiation with energy enough to promote the photogeneration of charge carriers, electrons (e^−^) and holes (*h^+^*) will be created in CB and VB, respectively. The dopant will contribute, with their *3d* bands, to boost the availability of such photo-generated charge carriers. In the CB, electrons will be in charge, considering the presence of adsorbed O_2_, to produce oxidant radicals, including O_2_^•−^ (−0.046 eV) and H_2_O_2_ (0.682 eV). Meanwhile, the photogenerated holes (*h^+^*) in the VB will be able to react with OH^−^ or H_2_O to generate •OH radicals. The overall action of *h^+^* and all the other oxidant species produced, including •OH and O_2_^•−^, will be responsible for the photocatalytic pollutant(s) oxidation processes.

Likewise, in samples containing Co and Fe, and considering their redox potentials, these two metallic species will simultaneously contribute for the creation of additional energy bands within TNW forbidden zone. These levels will act as electron or hole traps and consequently they will have a (positive) impact in charge carriers’ lifetime. Moreover, the simultaneous occurrence of a photo-Fenton process, can be achievable due to the co-existence of ionic Fe and Co in the TNW structure. 

In fact, for semiconducting materials containing Fe, the formation of H_2_O_2_ due to the O_2_ reduction has been reported [58]. Therefore, with the o-existence of H_2_O_2_ and Fe^3+^/Fe^2+^, the ideal conditions for photo-Fenton reaction to take place will be created (Equation (1)). This process will be responsible for an improved formation of •OH radicals, which in turn will be responsible for the degradation of contaminants.

Fe^2+^ + H_2_O_2_ → Fe^3+^ + •OH + OH^−^ (1)

Although Fe^2+^ assists as a Fenton-catalyst, it is well established that the kinetics of the Fe^3+^ → Fe^2+^ reaction is extremely slower if compared to the rate of Fe^2+^ → Fe^3+^ oxidation [11]. This kinetic constraint can limit its performance during photo-Fenton process. However, in the CoFeTNW material prepared in this work, the presence of Co also contributes to minimize this negative impact. In fact, Co^2+^ has been suggested as a promising Fenton-like catalyst mainly for organic contaminants removal. With the Co^2+^/Co^3+^ pair, the formation of •OH radicals, attributed to the redox reaction involving Co^2+^ and H_2_O_2_ species (Equation (2)) has been described in literature [43].

Co^2+^ + H_2_O_2_ → Co^3+^ + OH^−^ + •OH (2)

During the photo-activation of a material containing both Fe and Co, like CoFeTNW, the reduction of Co^3+^ → Co^2+^ can be due to the Fe^2+^ oxidation to Fe^3+^ (*E*^0^_Fe3+/Fe2+_ = +0.77 eV), since this is a thermodynamically favorable process (Equation (3)), or directly by the electrons from the CB (Equation (4)) [58].

Fe^2+^ + Co^3+^ → Fe^3+^ + Co^2+^, *E*^0^ = 1.04 eV (3)

Co^3+^ + 1e^−^ → Co^2+^, *E*^0^ = 1.81 eV (4)

The VB energy of the CoFeTNW sample (3.12 eV, Table S1), agrees with the oxidation, by the photo-generated *h^+^*, of the OH^−^or H_2_O to produce •OH radicals (Figure 8). 

The CB energy of such sample (0.64 eV, Table S1) is not compatible with the O_2_/O_2_^•−^ reduction (−0.046 eV) and therefore the *e−* will react with Fe^3+^ to produce Fe^2+^ (0.77 eV). The formation of H_2_O_2_, via O_2_ reduction (0.682 eV) with the consequent •OH production, will also occur.

These results and discussion seem to indicate that the co-doping (for instance with Fe and Co) can be a promising procedure to fabricate alternative and multi-tasking catalysts for, for instance, the degradation of emergent pollutants.

## 4. Conclusions

Titanate nanowires modified by Co and Fe co-doping were produced by a hydrothermal method. The structural and optical characterization by XRD and XPS strongly support that for CoTNW sample, the Co doping was accomplished by Ti^4+^/Co^2+^ replacement, in the TiO_6_ octahedra. For FeTNW and CoFeTNW powders, Fe and/or Co incorporation occurred by Ti^4+^/Co^2+^ or Ti^4+^/Fe^3+^ replacement, in interstitial positions or by incorporation of the ionic species between the TiO_6_ layers, via Na^+^ replacement. The catalytic performance of the prepared samples for the hydroxyl radical production and for ACAP photodegradation was investigated. Results show that CoFeTNW, demonstrate the best photocatalytic performance and it was also the best photocatalyst when a (ACAP+CAF) mixture was used. From the radical scavengers’ study, it was possible to conclude that the oxidant radicals in charge for ACAP removal were namely •OH and *h^+^*. A mechanism for the photo-activation of the CoFeTNW particles was proposed based on a synergetic combination of a heterogenous photo-Fenton reaction with a photocatalytic degradation process.

## Figures and Tables

**Table 1:** Relative atomic percentage of iron and cobalt incorporated in TNW, CoTNW, FeTNW and CoFeTNW samples and correspondent specific surface area.

Sample ID	Fe/Ti (%)	Co/Ti (%)	A_B.E.T._ (m^2^ g^−1^)
TNW	-	-	238.10
FeTNW	0.14	-	124.74
CoTNW	-	1.6	237.52
CoFeTNW	0.15	1.7	180.14

## Data Availability

Not applicable.

## Supplementary Materials

The following supporting information can be downloaded at: https://www.mdpi.com/article/10.3390/ma16052051/s1, Figure S1: Schematic representation of the synthesis pathway for TNW and related samples; Figure S2: TEM images of pristine TNW (a), FeTNW (b), CoTNW (c), and CoFeTNW (d) samples; Figure S3: Photoluminescence spectra of the prepared samples; Table S1: Bandgap (Eg), valence (EVB) and conduction (ECB) bands energies for TNW, CoTNW, FeTNW and CoFeTNW samples; Table S2: Ti binding energies for TNW, CoTNW, FeTNW and CoFeTNW samples; Table S3: Main fragments and correspondent intermediates identified by LC-ESI-QTOF-MS during ACAP photocatalytic degradation.
