# Supplementary material for: Enhancing Removal of Pollutants by Combining Photocatalysis and Photo-Fenton Using Co, Fe-Doped Titanate Nanowires
Source: Materials (Basel). 2023 Mar 1;16(5):2051. doi: 10.3390/ma16052051 (PMC10004198; doi:10.3390/ma16052051)
Supplement: Supplementary file 1 [file materials-16-02051-s001.zip › materials-2222831-supplementary.pdf]

Enhancing the removal of pollutants by combining photocatalysis and photo-Fenton using Co, Fe-doped titanate nanowires

B.T. Barrocas,[1] R. Osawa,[2] M. Conceição Oliveira,[3] O. C. Monteiro[1,*]

[1] Centro de Química Estrutural, Institute of Molecular Sciences, Departamento de Química e Bioquímica, Faculdade de Ciências, Universidade de Lisboa, Campo Grande, 1749-016 Lisboa, Portugal
[2] FT-ICR and Structural Mass Spectrometry Laboratory, MARE—Marine and Environmental Sciences Centre, Faculdade de Ciências, Universidade de Lisboa, 1749-016 Lisboa, Portugal
[3] Centro Química Estrutural, Institute of Molecular Sciences, Instituto Superior Técnico, ULisboa, 1049-001 Lisboa, Portugal
* Correspondence: ocmonteiro@ciencias.ulisboa.pt

Electronic supplementary information

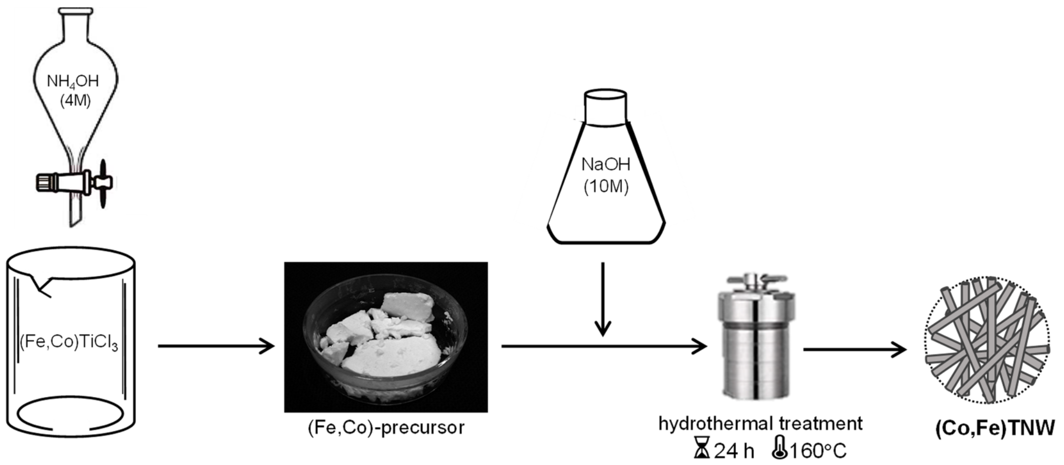

Figure S1. Schematic representation of the synthesis pathway for TNW and related samples.

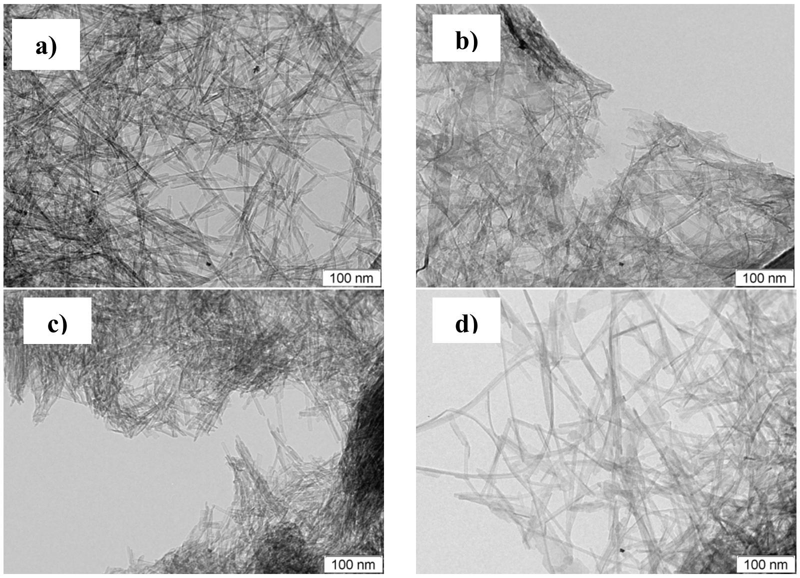

Figure S2. TEM images of pristine TNW (a), FeTNW (b), CoTNW (c), and CoFeTNW (d) samples.

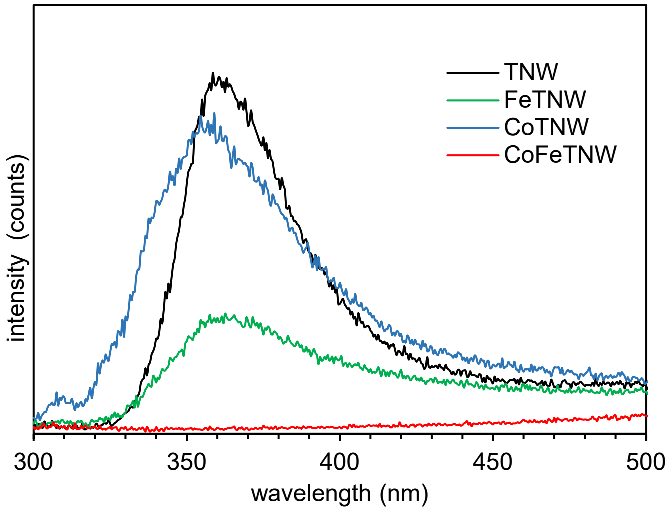

Figure S3. Photoluminescence spectra of the prepared samples.

Table S1. Bandgap (E_g), valence (E_{VB}) and conduction (E_{CB}) bands energies for TNW, CoTNW, FeTNW and CoFeTNW samples.

Sample ID	E_g (eV)	E_{VB} (eV)	E_{CB} (eV)
TNW	3.28	2.95	-0.33
FeTNW	2.97	2.95	-0.02
CoTNW	2.86	2.89	0.03
CoFeTNW	2.34	2.98	0.64

Table S2. Ti binding energies for TNW, CoTNW, FeTNW and CoFeTNW samples.

Sample ID	Ti binding energy (eV)		ΔE (eV)
	$2p_{3/2}$	$2p_{1/2}$	
TNW	458.641	464.441	5.8
FeTNW	458.478	464.678	6.2
CoTNW	458.360	464.660	6.3
CoFeTNW	458.494	464.594	6.1

Table S3. Main fragments and correspondent intermediates identified by LC-ESI-QTOF-MS during ACAP photocatalytic degradation.

Compound	Formula $[M + H]^+$	Experimental mass (m/z)	Mol.wt.	Structure
**Acetaminophen**	$C_8H_{10}NO_2$	152.07	151	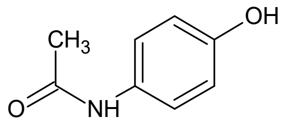
**ACAP-1**	C_6H_8NO	110.06	109	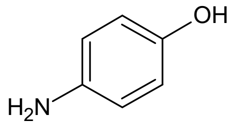
**ACAP-2**	$C_{14}H_{12}NO_5$	274.07	273	--
**ACAP-3**	$C_8H_{10}NO_4$	184.06	183	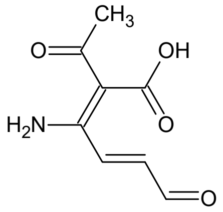
**ACAP-4**	$C_8H_{10}NO_3$	168.07	167	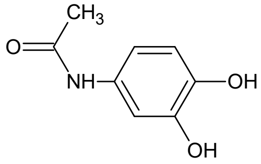
**ACAP-5**	$C_{11}H_{12}NO_4$	222.08	221	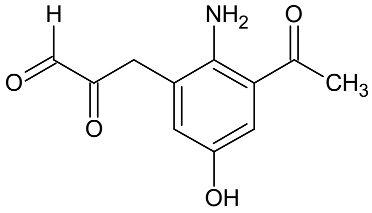
